# Exploring the mental health challenges of children with autoimmune thyroiditis

**DOI:** 10.1186/s12887-025-06109-2

**Published:** 2025-10-03

**Authors:** Yomna Ahmed Hosni, Marise Abdou, Mennat-Allah Tarek, Basant Ahmed Abd El-Alim

**Affiliations:** 1 The Diabetes Endocrine and Metabolism Pediatric Unit, Abo ELReesh Children’s Hospital, Faculty of Medicine, Cairo University, Cairo, Egypt; 2 Department of Pediatrics, Lecturer of Pediatrics, Member of The Diabetes Endocrine and Metabolism Pediatric Unit (DEMPU), Faculty of Medicine, Cairo University, Cairo, Egypt; 3 Department of Pediatrics, Member of The Diabetes Endocrine and Metabolism Pediatric Unit (DEMPU), Faculty of Medicine, Cairo University, Cairo, Egypt; 4 Department of Pediatrics, Lecturer of Psychiatry, Faculty of Medicine, Cairo University, Cairo, Egypt

**Keywords:** Autoimmune thyroiditis, Pediatrics, Strengths and difficulties, Health-related quality of life

## Abstract

**Background:**

Autoimmune thyroid disease is a common autoimmune disorder affecting the pediatric age group with a wide array of clinical presentations. Psychiatric symptoms may occur in association with autoimmune thyroiditis. This area has scarcely been explored in the pediatric age group. This study aimed to describe the clinical and laboratory features of autoimmune thyroid disease and to assess their behavioral characteristics and health-related quality of life.

**Methods:**

We retrospectively analyzed the records of 134 children up to the age of 18 years, who were following up in the Diabetes, Endocrine and Metabolism Pediatric Unit (DEMPU) at Abo El Rish El-Mounira Children’s Hospital, Kasr Al Ainy, Cairo University. Data collected from their medical records included clinical and laboratory parameters, including thyroid antibodies and thyroid ultrasound. Out of 134 patients’ records, 47 consented to participating in a psychiatric evaluation. Data obtained from theses evaluations was compared to that of 50 healthy matched control subjects.

**Results:**

The median age of the study group was 9.38 with IQR (7.16–11.68) years at the initial presentation of the illness. Goiter was the most prevalent presenting symptom in 26.1% of the cohort. Most of them (48.5%) were on thyroid replacement therapy (levothyroxine). Forty-seven of the patients consented to participate in a psychiatric evaluation. The patients had higher emotional and behavioral conduct problems scores than the controls with median and IQR [6 (4–80 and 4 (2–5)] versus [3 (1–5) and 2 (1–4)] with significant p-values of < 0.001 and 0.001 respectively on Strengths and Difficulties Questionnaire (SDQ). Additionally, the cases had lower physical well-being with a median of 3.25 and IQR (2.25–4.25) than the controls with a median of 4 and IQR (3.5–4.5) with a p-value of 0.014 on the KINDLR-parents’ version.

**Conclusions:**

Autoimmune thyroid disorders affecting the pediatric population can lead to emotional and conduct problems. Also, it affected their quality of life as they showed lower physical well-being scores. This should encourage earlier and thorough assessment of their psychological status and prompt management.

**Supplementary Information:**

The online version contains supplementary material available at 10.1186/s12887-025-06109-2.

## Background

The most prevalent cause of acquired thyroid dysfunction in children is autoimmune thyroid disorders (ATD), which include Hashimoto’s thyroiditis (HT) and Graves’ disease (GD) [1]. Autoimmune thyroid diseases are caused by the infiltration of autoreactive B and T lymphocytes of the thyroid gland and the presence of antithyroid antibodies. This will lead to variations in the thyroid function (GD: hyperthyroidism, HT: normal function, or subclinical/overt hypothyroidism [2].

Genetic components play a significant role in the occurrence of autoimmune diseases. In addition, maternal and paternal inheritance contributes to autoimmune thyroid diseases as more than 50% of children had a positive family history [3]. Autoimmune thyroid diseases can present at any age but usually at puberty [4]. Females tend to be affected more than males [5].

Many research and reviews have focused on the psychiatric evaluation of people with various forms of hyperthyroidism, including normal, overt, or subclinical thyroid hormone levels, in conjunction with anti-thyroid medications (ATDs) or thyroidectomy. Beyond the fundamental symptoms of thyrotoxicosis and other associated neuropsychiatric illnesses and disorders, these studies confirmed the incidence of more serious somatic symptoms. According to literature reviews, the prevalence of psychiatric symptoms associated with autoimmune thyroiditis varies greatly [6, 7]. For example, a systematic review and meta-analysis conducted by Slegmann et al. showed that autoimmune thyroiditis is associated with depression and anxiety disorders [8]. Research evaluating behavior in children with thyrotoxicosis is limited.

This study aimed to describe the clinical and laboratory features of autoimmune thyroid disease in our tertiary care center and assess their health-related quality of life and the strengths and difficulties that face them.

## Methods

This study was carried out in Diabetes, Endocrine and Metabolism Pediatric Unit (DEMPU) at Abo El Rish El-Mounira Children’s Hospital, Kasr Al Ainy, Cairo University. Medical records of children following up in the clinic of thyroid disorders were thoroughly reviewed, and data from charts of patients with autoimmune thyroiditis were collected. The patients’ files dated back to 2016. The study took place from July 2023 till January 2025. Patients up to 15 years of age were included who were presenting overt hypothyroidism, subclinical hypothyroidism, hyperthyroidism, or euthyroidism. In patients with negative thyroid anti-bodies, they were included based on clinical grounds or by ultrasound appearance. Charts of patients with other thyroid diseases, such as congenital hypothyroidism, were excluded. Informed consent was obtained from the participants and/or their parents.

The following data were collected from the Medical records: the age of the patients at diagnosis, family history of autoimmune thyroiditis, the patients’ weight, height, body mass index (BMI), and their SDS, the first thyroid-stimulating hormone (TSH), Free Triiodothyronine (FT3), Free thyroxine (FT4), thyroid antibodies (Anti-peroxidase and anti-thyroglobulin antibodies), and thyroid ultrasound and scan available.

Control group: Fifty healthy age and sex-matched children were enrolled from the general pediatric clinic after explaining the study’s aim and steps. Specifically, we clarify that the children in the control group were recruited from the pediatric outpatient clinic during routine check-ups or visits for minor, self-limiting conditions such as mild upper respiratory tract infections. These children were not diagnosed with any chronic medical, neurological, or psychiatric disorders, and they were not on any long-term medications. We ensured that they were developmentally appropriate for their age and exhibited no clinical signs suggestive of mental health concerns.

Medical records of 134 children were collected, and 47 consented to psychiatric evaluation using the Strengths and Difficulties Questionnaire (SDQ) and KINDLER-parents’ version used by Goodman et al., and Ravens-Sieberer et al., respectively [9, 10]. (Supplementary material 1,2) These patients were in an euthyroid state with variable durations.

The Strengths and Difficulties Questionnaire (SDQ) is a short psychological screening questionnaire for children aged 2 to 17. Each version of the SDQ asks about approximately 25 characteristics, some positive and some negative. These 25 items are grouped into five scales: emotional symptoms, peer relationship problems, conduct problems, prosocial behavior, hyperactivity/inattention, and total difficulties score. The higher scores indicate more difficulties [9].

KINDLR – parents’ version (7 to 17-year-olds) Health-Related Quality of Life (HRQoL) was assessed with the KINDL-R. The KINDL-R is a widely used self- or parent-reported questionnaire with strong psychometric properties. It includes 24 items that assess six domains of HRQoL (physical well-being, self-esteem, emotional well-being, family, friends, and daily functioning) using a five-point Likert scale (1–5). The total score can be calculated and converted to a value between 0 and 100. An improved quality of life is indicated with higher scores [10].

All questionnaires were administered in Arabic, which is the native language of the participating children. We used validated Arabic versions of the assessment tools to ensure linguistic and cultural appropriateness, as well as the reliability and validity of the data collected.

### Ethical approval

The study was designed as retrospective in nature, and we obtained complementary ethical approval from the institutional ethical committee to contact a subgroup of eligible participants and invite them to undergo psychiatric assessment using the relevant questionnaires. The study was approved by the Institutional Review Board of Cairo University and followed the Helsinki Declaration (A-28-2024).

### Statistical analysis

The statistical software for the social sciences (SPSS), version 28 (IBM Corp., Armonk, NY, USA) was used for coding and data entry. For quantitative data, the mean, standard deviation, median, minimum, and maximum were utilized for data summarization; for categorical data, the frequency (count) and relative frequency (%) were used. The non-parametric Mann-Whitney test was utilized for quantitative variables comparison [11]. The Spearman correlation coefficient was used to analyze correlations between quantitative variables [12]. Statistical significance was defined as P-values below 0.05.

## Results

This retrospective study included 134 participants diagnosed with autoimmune thyroid disorders, 103 (76.9%) females, and 31 (23.1%) males, who were following up at the Diabetes, Endocrine and Metabolism Pediatric Unit (DEMPU) at Abo El Rish El-Mounira Children's Hospital, Kasr Al Ainy, Cairo University.

The median age of the studied group was 9.38 and IQR (7.16- 11.68) years at the initial presentation of the illness. Forty-seven participants who took part in the psychiatric assessment had a median age of 12 and IQR (10.00–14.80) years. Their median duration of illness was 2.72 and IQR (1.93–4.25) years. Sixty-five (48.5%) of the participants were on thyroid replacement therapy (levothyroxine), 46 (34.3%) were on antithyroid medications, carbimazole, and 4 (3%) received both treatments. Six (4.5%) children were euthyroid and were not prescribed medications, and 6 (4.5%) patients developed euthyroidism during follow-up (as data stated in the patients’ files).. Positive consanguinity was reported in 29.9% of the children, and 34.3% had a positive history of autoimmune thyroid disorders (Table 1).

**Table 1:** Characteristics of the examined files of the 134 patients

		Median (IQR) and Count (%)
Age at diagnosis (years) (*n* = 134)		9.38 (7.16–11.68) *
Current age for psychiatric assessment(years) (*n* = 47)		12.00 (10.00–14.80) *
Duration of illness (years) (*n* = 47)		2.72 (1.93–4.25) *
Weight (kg) (*n* = 112)		31.00 (23.25–38.00) *
Weight (SDS) (*n* = 112)		−0.30 (−1.10–0.70) *
Height (cm) (*n* = 112)		132.00 (121.00–143.00) *
Height (SDS) (*n* = 112)		−0.50 (−1.50–0.40) *
BMI (*n* = 106)		16.50 (14.95–20.40) *
BMI (SDS) (*n* = 112)		1.10 (0.50–1.80) *
Presenting symptom	**Goiter**	35 (26.1%) **
	**Goiter and hyperthyroid symptoms**	11 (8.2%) **
	**Goiter and hypothyroid symptoms**	3 (2.2%) **
	**Hyperthyroid symptoms**	32 (23.9%) **
	**Hypothyroid symptoms**	18 (13.4%) **
	**Accidentally discovered**	1 (0.7%) **
	**Not stated**	34 (25.3%) **
Treatment	**Eltroxin**	65 (48.5%) **
	**Carbimazole**	46 (34.3%) **
	**Carbimazole and eltroxin**	4 (3.0%) **
	**On treatment and stopped**	6 (4.5%) **
	**No treatment**	6 (4.5%) **
	**Not stated**	7 (5.2%) **
Consanguinity	**Positive**	40 (29.9%) **
	**Negative**	65 (48.5%) **
Family history of autoimmune thyroiditis	**Yes**	46 (34.3%) **
	**No**	56 (41.8%) **
	**Not stated**	32 (23.9%) **

Data presented as median (IQR)* and count (percentage)**

*SDS* standard deviation score, *IQR* Interquartile range, *BMI* body mass index

Over half of the patients had positive anti-TPO Abs (80.43%), and 64.04% had positive anti-TG Abs with a median and IQR of 161.00 (64.00 - 290.00) and 130.60 (16.27 - 293.00, respectively. Regarding the initial US done at the presentation, 76.4% of the patients had goiter, 87.5% with a heterogenous pattern, 91.1% showed increased vascularity, and 9 patients had pseudo-nodules. During follow-up, 50% of patients had goiter with a heterogenous texture and an increase in vascularity. Regarding the thyroid scan, 39 patients had goiter, one with heterogenous texture, and around two-thirds with increased vascularity. (Table 2).

**Table 2:** Clinical and laboratory data of the study group as collected from the files

Investigations		Median (IQR) and Count (%)
FT3		4.43(2.99–9.10) *
TT3		177.0 (36.70–299.00) *
FT4		1.76 (0.70–4.35) *
TT4		7.17 (1.50–11.30) *
TSH		2.12 (0.02–15.53) *
Anti-TPO Abs	Positive	74 (55.22%)**
	Negative	17 (12.69%)**
	Not stated	43 (32.09%)**
Anti-TGs Abs	Positive	58 (43.28%) **
	Negative	34 (25.37%) **
	Not stated	42 (31.34%)**
Thyroid Ultrasound		
Goiter (Initial)	Yes	55 (76.4%) **
	No	17 (23.6%) **
Thyroid texture (Initial)	Homogenous texture	5 (12.5%) **
	Heterogenous texture	35 (87.5%) **
Thyroid vascularity (Initial)	Average	4 (8.9%) **
	Increased	41 (91.1%) **
Thyroid pseudo-nodules(Initial)	Yes	9 (100.0%) **
	No	0 (0.0%) **
Goiter (follow up)	Yes	3 (50.0%)
	No	3 (50.0%)
Thyroid texture (follow up)	Homogenous texture	0 (0.0%)
	Heterogenous texture	7 (100.0%)
Thyroid vascularity (follow-up)	Average	0 (0.0%)
	Increased	5 (100.0%)
Thyroid scan		
Goiter	Yes	39 (97.5%) **
	No	1 (2.5%) **
Thyroid uptake	Normal	9 (23.1%) **
	Increased	30 (76.9%) **
	Decreased	0 (0.0%) **
Thyroid texture	Homogenous	8 (88.9%) **
Thyroid uptake in the thyroid scan		10.65 (3.30–20.70) *

Data presented as median (IQR)* and count (percentage)**

*IQR* Interquartile range, *FT3* Free Triiodothyronine, *TT3* Total Triiodothyronine,* FT4* Free thyroxine, *TT4* Total thyroxine, *TSH* thyroid-stimulating hormone,* Anti-TPO Ab*s Anti-peroxidase antibodies, *Anti-TG Abs* Anti-thyroglobulin antibodies

Out of 134 participants, 47 agreed to take part in 2 questionnaires assessing health-related quality of life and strengths and difficulties that they face. The mean age of that group was 12.53 ± 3.04 years; 15 (31.91%) were males and 32 (68.08%) were females. Seven (14.9%) had a positive family history of psychiatric illness. They were compared to 50 controls who were age and sex-matched with a mean age of 11.46 ± 3.16 years, 19 (38%) were males and 31 (62%) were females, and 12 (24%) had a family history of psychiatric illness. The patients were euthyroid at the time they took the questionnaires, with a median duration of 6 months and IQR (4-12) months.

The following table shows the results of the Strengths and Difficulties Questionnaire, the cases had higher emotional and conduct behavioral disorder scores than the controls with median and IQR [6 (4-80 and 4 (2-5)] versus [3 (1-5) and 2 (1-4)] with significant p-values of<0.001 and 0.001 respectively. Also, the cases had a higher prosocial score than the controls with median and IQR [10 (8-10)] versus [7 (6-7)] with a significant p-value of <0.001. (Table 3).

**Table 3.** Strengths and Difficulties Questionnaire (SDQ) of cases versus controls (n=47)

	Controls		Cases		
	Median	IQR	Median	IQR	P value
Emotional problems score	3	1–5	6	4–8	< 0.001*
Conduct problems score	2	1–4	4	2–5	0.001*
Hyperactivity score	4	2–6	4	2–7	0.515
Peer problems score	2	1–3	3	1–4	0.452
Prosocial score	7	6–7	10	8–10	< 0.001*
Parents impact score	0	0–2	3	0–4	0.002*
Total difficulties score	13	8–15	17	13–22	0.001*

Data were presented as median and IQR

According to the KINDLR – parents’ version (7 to 17-year-olds) Health-Related Quality of Life (HRQoL) of cases versus controls, the cases had lower physical well-being with a median of 3.25 and IQR (2.25-4.25) than the controls with a median of 4 and IQR (3.5-4.5) with a p-value of 0.014. While the cases had higher school performance with a median of 4 and IQR (3-5) than the controls with a median of 3.25 and IQR (3-3.25) with a p-value of 0.003. (Table 4).

**Table 4:** KINDLR – parents’ version (7 to 17-year-olds) Health-Related Quality of Life (HRQoL) of cases versus controls (n=47)

	Controls		Cases		
	Median	IQR	Median	IQR	P value
Physical Well-being	4	3.5–4.5	3.25	2.25–4.25	0.014*
Emotional wellbeing	4.25	3.5–4.50	3.75	3.25–4.50	0.334
Self-esteem	3.75	3–4	4	3-4.50	0.275
Family	4	3.50–4.50	4	3-4.50	0.716
Friends	4	3.25–4.25	4.25	3–5	0.360
School	3.25	3-3.25	4	3–5	0.003*
Total quality of life score	3.83	3.46–4.13	4	3-4.25	0.965

Data were presented as median and IQR

## Discussion

 The current study retrospectively examined 134 medical records of patients diagnosed with autoimmune thyroid disorders, 103 (76.9%) females, and 31 (23.1%) males, who were following up in the Diabetes, Endocrine and Metabolism Pediatric Unit (DEMPU) at Abo El Rish El-Mounira Children's Hospital, Kasr Al Ainy, Cairo University. The female/male ratio was 3.32/1, similar to a study by Karakaya et al. with a ratio of 3.5/1 [13] This was also demonstrated by similar studies with females having a higher ratio than males [14–18].

Goiter was the most common presentation in our patients with 26.1% followed by hyperthyroid symptoms (23.9%) and hypothyroid symptoms (13.4%). Also, some patients had both goiter and hyperthyroid or hypothyroid symptoms with 8.2% and 2.2% respectively. Similarly, a recent study conducted in Turkey showed goiter as the most common presentation with a percentage of 37.9% [13]. This aligned with another study conducted in China with goiter occurring in 21.1% of the patients [19]. Demirbilek et al., and Tunç et al. showed similar results with 54.9% and 43.5% of the cases of Hashimoto’s thyroiditis having goiter respectively [14, 18].

 Cases presenting with hyperthyroidism and hypothyroidism were lower in some studies. A retrospective study conducted retrospectively on 119 cases between 2013-2020 described 21.8% having overt hypothyroidism and 4.2% having overt hyperthyroidism [13]. In addition, a study including 108 patients showed 16.6% having overt hypothyroidism and 3.7% having hyperthyroidism [14]. This also was the case in a study conducted on 19 patients with Hashimoto thyroiditis below the age of 3 years, which showed 16.6% having overt hypothyroidism (16.6%), and 3.7% having hyperthyroidism [19].

 According to published research, between 40 and 70 percent of children with Hashimoto's thyroiditis use L-thyroxine [20–23]. Levothyroxine was the most common medication taken in our patients in 46 (34.3%) of them. Our results were comparable to the results reported by Tunc et al, with 43.5% [14].

Hashimoto’s thyroiditis has a genetic predisposition. According to cohort research conducted in India, Hashimoto's thyroiditis was nine times more likely to affect first-degree relatives of individuals with the condition than it was in the general adult population [24]. According to a different study, mothers with Hashimoto’s thyroiditis were 32 times more likely to pass on autoimmune thyroiditis to their children [25]. In our study, 34.3% of children had a positive history of autoimmune thyroiditis. This percentage was comparable to those reported by Karakaya et al., Tunç et al., Tang et al., Tuhan et al., and Wasniewska et al., with 36.1%, 59%, 47.4%, 47.5%, and 31.6% respectively [13, 14, 19, 26, 27].

 Regarding the thyroid antibodies, 55.22% of children had positive anti-TPO Abs and 43.28% had positive anti-TG Abs. Our findings were lower than those reported by Tang et al. with anti-TPO Abs and anti-TG Abs being positive in 89.5% of their cohort, Tunç et al. with anti-TPO Abs positive in 95% and anti-TG Abs being positive in 87% of their cohort, and Karakaya et al, with anti-TPO positive in 87.5% and anti-TG Abs positive in 68.9% of their cohort [13, 14, 19]. This might be explained that antibodies were not done in all of our patients due to high financial cost.

 As extracted from the records of the initial thyroid ultrasound done at the presentation, 76.4% of the patients had a goiter, 87.5% with a heterogenous pattern, 91.1% with increased vascularity, and 9 patients had pseudo-nodules. These findings were variable in several studies. Karakaya et al stated that 37.9% of the patients had a goiter and 11.1% had nodules in the thyroid ultrasound [13]. The percentage of goiter detected by thyroid ultrasound was 53.7% in the research carried out by Tang et al. [19]. Whereas, Tunç et al., reported goiter in 53.7% of cases with 88% having heterogenous parenchyma and 12% having homogenous [14].

 Out of the 134 patients’ reports, 47 patients agreed to undergo psychiatric evaluation using the Strengths and Difficulties Questionnaire (SDQ) and Kiddle Quality of Life questionnaire. They were compared to 50 Healthy matched control children. Their SDQ assessment revealed a statistically significant difference between both groups regarding the emotional problem score, which was developed to evaluate mood, worry, fears, and mood-related somatic complaints. In the current study, the cases show higher emotional problem scores, which comes in line with a study conducted on patients with autoimmune thyroiditis in the euthyroid state, which supported that the risk of depressive and anxiety disorders was related to thyroiditis [28]. However, Hirtz and his team (2020) found that thyroid disease did not significantly affect the emotional state of children and adolescents who participated in their study [29].

The relation between autoimmune thyroid disorders and the presence of mood disturbances and emotional problems may be attributed to that autoimmune activity that stimulates the secretion of pro-inflammatory cytokines, which cause neuroendocrine and neurotransmitter alterations. The elevated levels of pro-inflammatory cytokines may be related to the activation of central inflammation [30]. Peripherally generated cytokines can cross the blood-brain barrier and impact neurogenesis in hippocampal neurons, which is thought to play a significant role in the pathogenesis of mood disturbances [31, 32].

Conduct problems in children and adolescents are a serious concern because of their relationship with long-term negative consequences such as antisocial behaviour and substance abuse. In our study, children and adolescents with autoimmune thyroiditis showed significantly higher scores in the conduct problem subscale in comparison to the control group which is in congruence with a study that assessed the effect of thyroid troubles especially autoimmune thyroiditis during pregnancy on adolescent offspring and they claimed an increase in oppositional defiant disorder and conduct behaviour among them [33]. In addition, there is a study in the adult population found a correlation between thyroid hormone levels and impulsivity and aggression in those who committed crimes [34]. On the contrary, a Turkish study reported that their study’s control group unexpectedly had higher scores on emotional and conduct problems subscales in comparison to the patients’ group [35].

Furthermore, our study discovered that the cases group had significantly more prosocial behaviour than the controls. Unlike other studies conducted by Berberoğlu & Görker, found no difference between the 2 groups regarding prosocial behaviour [36]. The explanation of increased prosocial behavior may be based on the concept"Altruism born of suffering"as prosocial activity stemming from a person's bad experiences that make them more capable of giving and make them feel more responsible for preventing the pain of others [37, 38]. The negative experiences that children and adolescents with autoimmunethyroiditis may have include recurrent venipunctures for thyroid function measurement, medication adherence, and changes in appearance such as weight gain and hair loss. Moreover, regular clinic and hospital visits for follow-up and watching other children's sufferings act as important factors in their prosocial behaviour development.

 Regarding the quality of life, there were no statistically significant differences between cases and control groups except in physical well-being as patients showed lower physical well-being scores which are in contrast with research carried out by Uysal & Ayhan (2016), they found that autoimmune thyroiditis affects the subjective health status independent of thyroid function [39] (Uysal & Ayhan, 2016), this may be due to oxidative stress theory that can persist in autoimmune thyroiditis patients, causing cellular damage, decreased energy generation and exacerbating physical complains [40].

In the school activity domain, surprisingly, patients showed higher scores than the control group, which may be related to the high care and support provided by parents and teachers to help them cope with their peers.

### Limitation

Our study was a retrospective study using patients'files. Also, less than half of the patients agreed to take part in the psychiatric assessment, so a relatively small sample underwent psychiatric evaluation. In addition, TSH receptor antibodies were not done as they are of limited availability and expensive. Also, not all patients did Anti-TPO and anti-TG antibodies; we were only able to diagnose autoimmune thyroiditis but not classify them to Hashimoto thyroiditis or Graves’ disease. In addition, some had negative antibodies, so diagnosis was made on strong clinical suspicion and ultrasonographic findings. Antibodies will be done for these patients in follow-up.

### Recommendations

We recommend early and thorough psychiatric assessment of children with autoimmune thyroiditis and their prompt management and long-term follow-up. Also, to conduct this study on a larger scale and for a longer duration of follow-up.

## Conclusion

Autoimmune thyroiditis is a common autoimmune disease affecting children. It can present with a vast range of clinical presentations. According to our findings, children with autoimmune thyroiditis had emotional and conduct problems and lower physical well-being scores.

## Supplementary Information

Supplementary material 1.

Supplementary material 2.

## Data Availability

The datasets used and/or analysed during the current study are available from the corresponding author on reasonable request.

## Abbreviations

DEMPU: Diabetes, Endocrine and Metabolism Pediatric Unit
SQR: Strengths and Difficulties Questionnaire
ATD: Autoimmune thyroid disorders
HT: Hashimoto's thyroiditis
GD: Graves' disease
ATDs: Anti-thyroid medications
BMI: Body mass index
TSH: Thyroid-stimulating hormone
FT3: Free Triiodothyronine
FT4: Free thyroxine
HRQoL: Health-Related Quality of Life
SPSS: Statistical software for the social sciences
Anti-TPO Abs: Anti-peroxidase antibodies
Anti-TG Abs: Anti-thyroglobulin antibodies
IQR: Interquartile range
TT3: Total Triiodothyronine
