# Supplementary material for: Exploring the mental health challenges of children with autoimmune thyroiditis
Source: BMC Pediatr. 2025 Oct 3;25:751. doi: 10.1186/s12887-025-06109-2 (PMC12492639; doi:10.1186/s12887-025-06109-2)
Supplement: Supplementary file 1 — Supplementary material 1. [file 12887_2025_6109_MOESM1_ESM.pdf]

ID: __ __ __ __ __

Quality of Life Questionnaire for Children

Kid- & Kiddo-KINDL
Parents' Questionnaire KINDL[R]

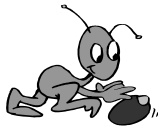

Dear Parent,

We really appreciate your taking the time to complete this questionnaire about your child's well-being and health-related quality of life.
Since it is a matter of **<u>your</u>** own assessment of your child's well-being, please complete the questionnaire yourself according to the instructions, i.e. without asking your child.

- ⇨ Read each question carefully.
- ⇨ Think about how your child has been feeling during the past week.
- ⇨ Put a cross in the box corresponding to the answer **<u>in each line</u>** that fits your child best.

For example:

During the past week ...	never	seldom	some-times	often	all the time
. ...my child has slept well	☐	☐	☐	☒	☐

My Child is a: ☐ Girl ☐ Boy

Age: ____ Years

You are: ☐ Mother ☐ Father ☐ Other _______________?

Date of fill out: __ / __ / __ (day / month / year)

1. Physical Well-being

	During the past week ...	never	seldom	some-times	often	all the time
1.	… my child felt ill	☐	☐	☐	☐	☐
2.	… my child had a headache or tummy-ache	☐	☐	☐	☐	☐
3.	... my child was tired and worn-out	☐	☐	☐	☐	☐
4.	... my child felt strong and full of energy	☐	☐	☐	☐	☐

2. Emotional Well-being

	During the past week ...	never	seldom	some-times	often	all the time
1.	... my child had fun and laughed a lot	☐	☐	☐	☐	☐
2.	... my child didn't feel much like doing anything	☐	☐	☐	☐	☐
3.	... my child felt alone	☐	☐	☐	☐	☐
4.	... my child felt scared or unsure of him-/ herself	☐	☐	☐	☐	☐

3. Self-esteem

	During the past week ...	never	seldom	some-times	often	all the time
1.	... my child was proud of him-/herself	☐	☐	☐	☐	☐
2.	... my child felt on top of the world	☐	☐	☐	☐	☐
3.	... my child felt pleased with him-/ herself	☐	☐	☐	☐	☐
4.	... my child had lots of good ideas	☐	☐	☐	☐	☐

4. Family

	During the past week ...	never	seldom	some-times	often	all the time
1.	... my child got on well with us as parents	☐	☐	☐	☐	☐
2.	... my child felt fine at home	☐	☐	☐	☐	☐
3.	... we quarrelled at home	☐	☐	☐	☐	☐
4.	... my child felt that I was bossing him/ her around	☐	☐	☐	☐	☐

5. Social Contacts

	During the past week ...	never	seldom	some-times	often	all the time
1.	... my child did things together with friends	☐	☐	☐	☐	☐
2.	... my child was liked by other kids	☐	☐	☐	☐	☐
3.	... my child got along well with his/ her friends	☐	☐	☐	☐	☐
4.	... my child felt different from other children	☐	☐	☐	☐	☐

6. School

	During the last week in which my child was at school ...	never	seldom	some-times	often	all the time
1.	... my child easily coped with schoolwork	☐	☐	☐	☐	☐
2.	... my child enjoyed the school lessons	☐	☐	☐	☐	☐
3.	... my child worried about his/her future	☐	☐	☐	☐	☐
4.	... my child was afraid of bad marks or grades	☐	☐	☐	☐	☐

Thank you for your co-operation!
