# Supplementary material for: Exploring the mental health challenges of children with autoimmune thyroiditis
Source: BMC Pediatr. 2025 Oct 3;25:751. doi: 10.1186/s12887-025-06109-2 (PMC12492639; doi:10.1186/s12887-025-06109-2)
Supplement: Supplementary file 2 — Supplementary material 2. [file 12887_2025_6109_MOESM2_ESM.pdf]

Scoring the Strengths & Difficulties Questionnaire for age 4-17 or 18+

The 25 items in the SDQ comprise 5 scales of 5 items each. It is usually easiest to score all 5 scales first before working out the total difficulties score. 'Somewhat True' is always scored as 1, but the scoring of 'Not True' and 'Certainly True' varies with the item, as shown below scale by scale. For each of the 5 scales the score can range from 0 to 10 if all items were completed. These scores can be scaled up pro-rata if at least 3 items were completed, e.g. a score of 4 based on 3 completed items can be scaled up to a score of 7 (6.67 rounded up) for 5 items.

Note that the items listed below are for 4-17-year-olds, but the scoring instructions are identical for the similarly-worded '18+' SDQ

Table 1: Scoring symptom scores on the SDQ for 4-17 year olds

	Not True	Somewhat True	Certainly True
**Emotional problems scale**			
ITEM 3: Often complains of headaches… *(I get a lot of headaches…)*	0	1	**2**
ITEM 8: Many worries… (*I worry a lot)*	0	1	**2**
ITEM 13: Often unhappy, downhearted… (*I am often unhappy….)*	0	1	**2**
ITEM 16: Nervous or clingy in new situations… (*I am nervous in new situations…)*	0	1	**2**
ITEM 24: Many fears, easily scared *(I have many fears…)*	0	1	**2**
**Conduct problems Scale**			
ITEM 5: Often has temper tantrums or hot tempers (*I get very angry)*	0	1	**2**
ITEM 7: Generally obedient… *(I usually do as I am told)*	**2**	1	0
ITEM 12: Often fights with other children… *(I fight a lot)*	0	1	**2**
ITEM 18: Often lies or cheats *(I am often accused of lying or cheating)*	0	1	**2**
ITEM 22: Steals from home, school or elsewhere *(I take things that are not mine)*	0	1	**2**
**Hyperactivity scale**			
ITEM 2: Restless, overactive… *(I am restless…)*	0	1	**2**
ITEM 10: Constantly fidgeting or squirming *(I am constantly fidgeting….)*	0	1	**2**
ITEM 15: Easily distracted, concentration wanders *(I am easily distracted)*	0	1	**2**
ITEM 21: Thinks things out before acting *(I think before I do things)*	**2**	1	0
ITEM 25: Sees tasks through to the end… *(I finish the work I am doing)*	**2**	1	0
**Peer problems scale**			
ITEM 6: Rather solitary, tends to play alone *(I am usually on my own)*	0	1	**2**
ITEM 11: Has at least one good friend *(I have one good friend or more)*	**2**	1	0
ITEM 14: Generally liked by other children *(Other people my age generally like me)*	**2**	1	0
ITEM 19: Picked on or bullied by other children… *(Other children or young people pick on me)*	0	1	**2**
ITEM 23: Gets on better with adults than with other children *(I get on better with adults than with people my age)*	0	1	**2**
**Prosocial scale**			
ITEM 1: Considerate of other people's feelings *(I try to be nice to other people)*	0	1	**2**
ITEM 4: Shares readily with other children… *(I usually share with others)*	0	1	**2**
ITEM 9: Helpful if someone is hurt… *(I am helpful is someone is hurt…)*	0	1	**2**
ITEM 17: Kind to younger children *(I am kind to younger children)*	0	1	**2**
ITEM 20: Often volunteers to help others… *(I often volunteer to help others)*	0	1	**2**

<u>Total difficulties score</u>: This is generated by summing scores from all the scales except the prosocial scale. The resultant score ranges from 0 to 40, and is counted as missing of one of the 4 component scores is missing.

<u>'Externalising' and 'internalising' scores</u>: The externalising score ranges from 0 to 20 and is the sum of the conduct and hyperactivity scales. The internalising score ranges from 0 to 20 and is the sum of the emotional and peer problems scales. Using these two amalgamated scales may be preferable to using the four separate scales in community samples, whereas using the four separate scales may add more value in high-risk samples *(see Goodman & Goodman. 2009 Strengths and difficulties questionnaire as a dimensional measure of child mental health. J Am Acad Child Adolesc Psychiatry 48(4), 400-403).*

<u>Generating impact scores</u>

When using a version of the SDQ that includes an 'impact supplement', the items on overall distress and impairment can be summed to generate an impact score that ranges from 0 to 10 for parent- and self-report, and from 0 to 6 for teacher-report.

Table 2: Scoring the SDQ impact supplement

	Not at all	Only a little	A medium amount	A great deal
**<u>Parent report:</u>**				
Difficulties upset or distress child	0	0	1	2
Interfere with HOME LIFE	0	0	1	2
Interfere with FRIENDSHIPS	0	0	1	2
Interfere with CLASSROOM LEARNING	0	0	1	2
Interfere with LEISURE ACTIVITIES	0	0	1	2
**<u>Teacher report:</u>**				
Difficulties upset or distress child	0	0	1	2
Interfere with PEER RELATIONS	0	0	1	2
Interfere with CLASSROOM LEARNING	0	0	1	2
**<u>Self-report report:</u>**				
Difficulties upset or distress child	0	0	1	2
Interfere with HOME LIFE	0	0	1	2
Interfere with FRIENDSHIPS	0	0	1	2
Interfere with CLASSROOM LEARNING	0	0	1	2
Interfere with LEISURE ACTIVITIES	0	0	1	2

Responses to the questions on chronicity and burden to others are not included in the impact score. When respondents have answered 'no' to the first question on the impact supplement (i.e. when they do not perceive themselves as having any emotional or behavioural difficulties), they are not asked to complete the questions on resultant distress or impairment; the impact score is automatically scored zero in these circumstances.

Cut-points for SDQ scores for age 4-17: original 3-band solution & newer 4-band solution

Although SDQ scores can be used as continuous variables, it is sometimes convenient to categorise scores. The initial bandings presented for the SDQ scores were 'normal', 'borderline' and 'abnormal'. These bandings were defined based on a population-based UK survey, attempting to choose cutpoints such that 80% of children scored 'normal', 10% 'borderline' and 10% 'abnormal'.

More recently a four-fold classification has been created based on an even larger UK community sample. This four-fold classification differs from the original in that it (1) divided the top 'abnormal' category into two groups, each containing around 5% of the population, (2) renamed the four categories (80% 'close to average', 10% 'slightly raised, 5% 'high' and 5% 'very high' for all scales except prosocial, which is 80% 'close to average', 10% 'slightly lowered', 5% 'low' and 5% 'very low'), and (3) changed the cut-points for some scales, to better reflect the proportion of children in each category in the larger dataset.

Note that these cut points have not been validated for use with the 18+ SDQ, so we suggest that it is safest to use continuous scores rather than categories for this measure

Table 3: Categorising SDQ scores for 4-17 year olds (not validated for 18+)

	Original 3-band categorisation			Newer 4-band categorisation			
	Normal	Borderline	Abnormal	Close to average	Slightly raised (/slightly lowered)	High (/Low)	Very high (very low)
**Parent completed SDQ**							
Total difficulties score	0-13	14-16	17-40	0-13	14-16	17-19	20-40
Emotional problems score	0-3	4	5-10	0-3	4	5-6	7-10
Conduct problems score	0-2	3	4-10	0-2	3	4-5	6-10
Hyperactivity score	0-5	6	7-10	0-5	6-7	8	9-10
Peer problems score	0-2	3	4-10	0-2	3	4	5-10
Prosocial score	6-10	5	0-4	8-10	7	6	0-5
Impact score	0	1	2-10	0	1	2	3-10
**Teacher completed SDQ**							
Total difficulties score	0-11	12-15	16-40	0-11	12-15	16-18	19-40
Emotional problems score	0-4	5	6-10	0-3	4	5	6-10
Conduct problems score	0-2	3	4-10	0-2	3	4	5-10
Hyperactivity score	0-5	6	7-10	0-5	6-7	8	9-10
Peer problems score	0-3	4	5-10	0-2	3-4	5	6-10
Prosocial score	6-10	5	0-4	6-10	5	4	0-3
Impact score	0	1	2-6	0	1	2	3-6
**Self-completed SDQ**							
Total difficulties score	0-15	16-19	20-40	0-14	15-17	18-19	20-40
Emotional problems score	0-5	6	7-10	0-4	5	6	7-10
Conduct problems score	0-3	4	5-10	0-3	4	5	6-10
Hyperactivity score	0-5	6	7-10	0-5	6	7	8-10
Peer problems score	0-3	4-5	6-10	0-2	3	4	5-10
Prosocial score	6-10	5	0-4	7-10	6	5	0-4
Impact score	0	1	2-10	0	1	2	3-10

Note that both these systems only provide a rough-and-ready way of screening for disorders; combining information from SDQ symptom and impact scores from multiple informants is better, but still far from perfect.
